# Magmatic history of the Oldest Toba Tuff inferred from zircon U–Pb geochronology

**DOI:** 10.1038/s41598-020-74512-z

**Published:** 2020-10-15

**Authors:** Hisatoshi Ito

**Affiliations:** Nuclear Risk Research Center, Central Research Institute of Electric Power Industry, Chiba, 270-1194 Japan

**Keywords:** Environmental sciences, Natural hazards, Solid Earth sciences

## Abstract

The magmatic history of the Oldest Toba Tuff (OTT), the second largest in volume after the Youngest Toba Tuff (YTT), northern Sumatra, Indonesia, was investigated using U–Pb zircon dating by LA-ICP-MS. Zircon dates obtained from surface and interior sections yielded ages of 0.84 ± 0.03 Ma and 0.97 ± 0.03 Ma, respectively. The youngest OTT zircon ages were in accordance with the ^40^Ar/^39^Ar eruption age of ~ 0.8 Ma, whereas the oldest zircon dates were ~ 1.20 Ma. Therefore, the distribution of zircon U–Pb ages is interpreted to reflect protracted zircon crystallization, suggesting that the estimated 800–2,300 km^3^ of OTT magma accumulated and evolved for at least 400,000 years prior to eruption. This result is comparable to the volume and timescales of YTT magmatism. The similarities of both magmatic duration and geochemistry between OTT and YTT may indicate that they are similar in size and that the caldera collapse that generated OTT might be much larger previously interpreted.

## Introduction

**Figure 1:**
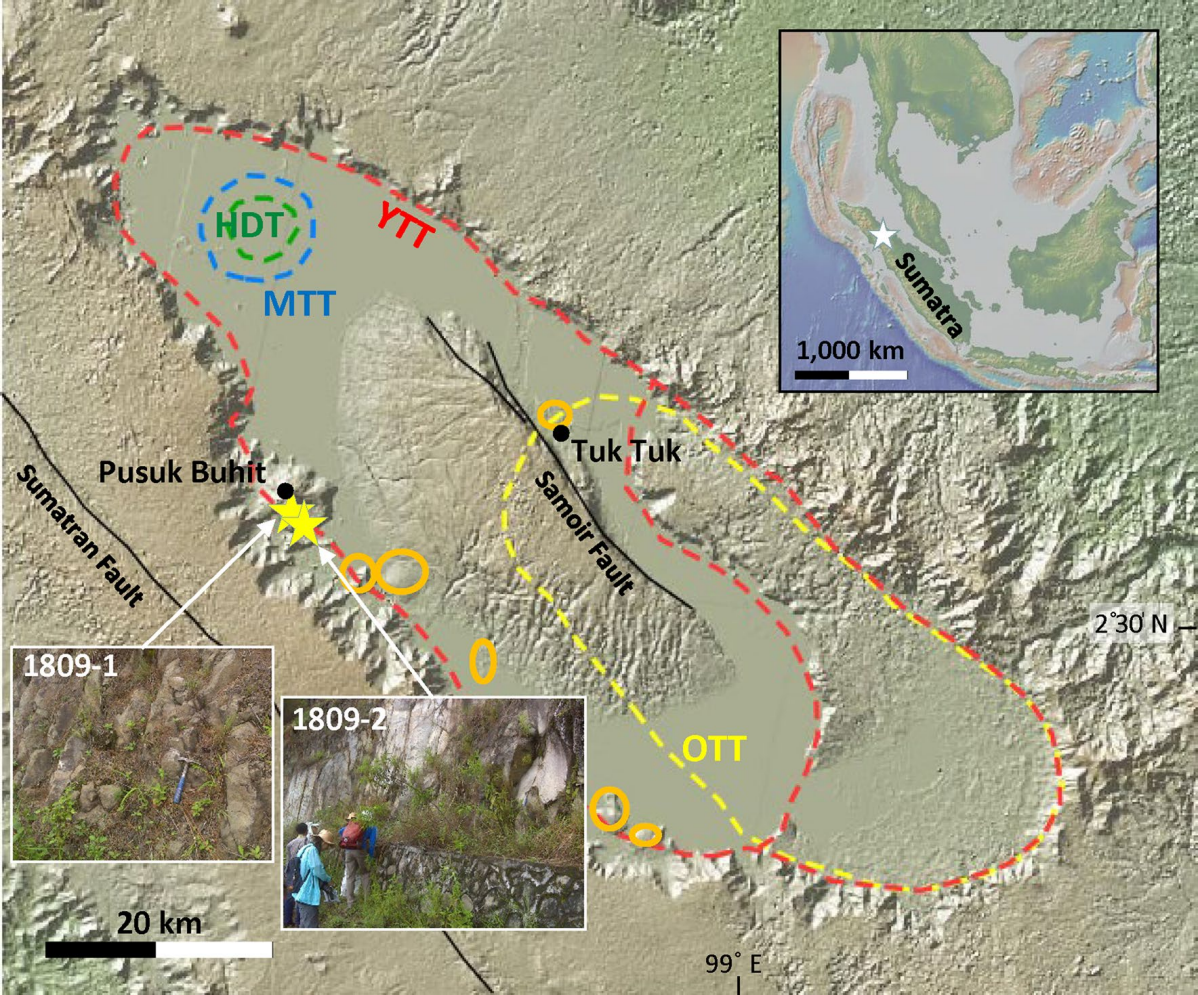
Map of the Toba Caldera Complex (TCC) (adapted from GeoMapApp; www.geomapapp.org) with sampling localities (yellow star). Inferred caldera locations are indicated by colored dashed lines: Haranggaol Dacite Tuff (HDT) = green, Oldest Toba Tuff (OTT) = yellow, Middle Toba Tuff (MTT) = blue, and Youngest Toba Tuff (YTT) = red. Post-caldera lava domes are outlined in orange circles (modified after Chesner^18^). Towns are indicated by black dots. Inset: Map of Sumatra with TCC marked with a white star.

The Toba Caldera Complex (TCC) (Fig. 1), northern Sumatra, Indonesia, is identified as the site of super-eruptions that may have severely affected the Earth’s climate and human evolution^1^. There were at least two super-eruptions at Toba in the Quaternary: The ~ 0.8 Ma Oldest Toba Tuff (OTT)^2–4^ and the ~ 0.074 Ma Youngest Toba Tuff (YTT)^5–7^. The magmatic evolution of the YTT has been previously well-described^7,8^ using allanite and zircon U–Pb geochronology, which showed that the YTT magma accumulated and evolved over a period of 500,000 years. On the contrary, the magmatic evolution of OTT has not been well-documented. Here, I applied U–Pb dating on zircon separated from OTT samples to better understand the timescales of magmatic evolution. The results reveal that OTT experienced hundreds of thousands of years of magmatic evolution before its cataclysmic eruption; an eruptive history very similar to YTT.

**Figure 2:**
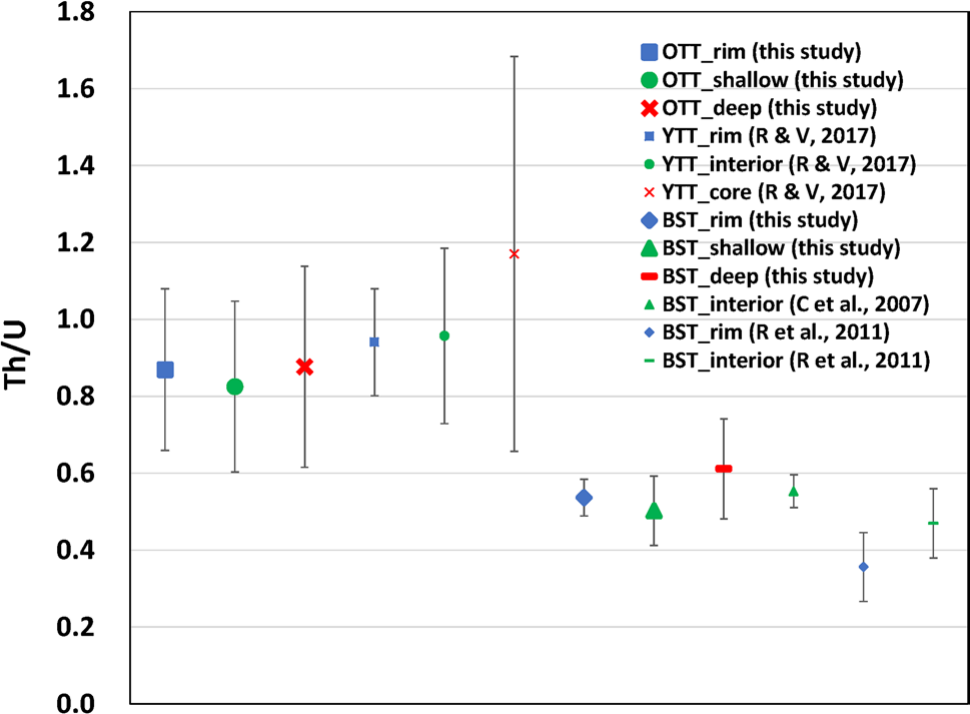
Mean zircon Th/U with 1 standard deviation for the Toba tuff units (OTT and YTT) and the Bishop Tuff (BST), Long Valley Caldera, USA. The Th/U remains at ~ 0.9 for the Toba tuffs throughout its long eruptive history (from ~ 1.2 Ma to ~ 0.1 Ma) and differs remarkably from that of the Bishop Tuff (~ 0.5), which reflects local crustal assimilation. Rim, shallow, deep, interior, and core indicate different depth sections of zircon grains. R & V, 2017: Reid and Vazquez^7^; C et al., 2007: Crowley et al.^43^; R et al., 2011: Reid et al.^44^. Data are shown in Supplementary Table S5.

Zircon U–Th–Pb geochronology and trace element compositions can provide powerful insight into the duration and frequency of magma assembly prior to a super-eruption^7^ (Fig. 2). Due to its small grainsize and refractory nature, zircon tends to have a strong affinity for the liquid phase and is a faithful recorder of magma evolution^9–12^. Reid and Vazquez^7^ showed that YTT zircons nucleated episodically and persisted over 500,000 years at the cool and wet eutectic conditions well before the ~ 0.074 Ma super-eruption based on U–Th–Pb ages measured by secondary ion mass spectrometry (SIMS) collected on both surface and interior of zircon crystals. Compared to SIMS, laser ablation-inductively coupled plasma-mass spectrometry (LA-ICP-MS) requires more volume to analyze a single zircon, hence it may be challenging to obtain ages from surfaces (or rims) from crystals with complex crystallization histories^13^. Here, I applied a new methodology to demonstrate that ages obtained on zircon rims using LA-ICP-MS are useful and I compare these with results obtained from deeper parts of zircon measured in the same analysis.

## Brief history of the Toba Caldera Complex and the OTT samples

The TCC is a 100 × 30 km topographic depression of several overlapping calderas that collapsed as a result of four major eruptions during the Quaternary^14,15^ (Fig. 1). It also is the largest resurgent Quaternary caldera on Earth^15–18^ and elongated in a NW–SE direction parallel to the active volcanic front of Sumatra. TCC has been the locus of silicic volcanic activity for at least 1.3 million years^19^. The cataclysmic ~ 0.074 Ma YTT produced 2,800 km^3^ (minimum volume) of non-welded to densely welded ignimbrite and associated ashfall deposit^5,6^, which exceeds the eruptive volume from the three preceding ignimbrites: the ~ 1.2 Ma (~ 35 km^3^) Haranggaol Dacite Tuff (HDT)^20^, the ~ 0.8 Ma (~ 800 to 2,300 km^3^) OTT^2,3^, and the ~ 0.5 Ma (≥ 60 km^3^) Middle Toba Tuff (MTT)^15^.

The voluminous YTT rhyolite was likely vented from a melt-rich subvolcanic reservoir located at a depth equivalent to 110–180 MPa^7,21^ (or 4–7 km, assuming a mean crustal density of 2.7 g cm^−3^). Judging by the size of the cauldron block at Lake Toba (Fig. 1) and the seismically imaged sill-like magmatic complex below it^22^, the associated intrusive volume is likely batholithic in scale^7^.

The location of eruptive vents for the second largest OTT eruption are not well constrained. Chesner^18^ suggested the OTT vented in the south of the present Toba Caldera (Fig. 1) based on the ignimbrite exposures in the southeastern part of the present caldera. Alternatively, Knight et al.^14^ suggested that OTT also vented from the northern part of the caldera based on a magnetic fabric investigation of the volcanic deposits.

Petrologic studies conducted at Toba^17,18^ are consistent with a silicic mush model^23^ and a compositionally zoned magma chamber^24^. Modeling results from these studies suggest that the quartz-bearing Toba tuffs and associated calderas represent the surface expression of a large granitoid batholith that episodically provides monotonous composition melts to shallow magma reservoirs^18^. All TCC samples have initial ^87^Sr/^86^Sr between 0.71333 and 0.71521^17,18^, suggesting a compositionally restricted source of the parental Toba magmas to the crust, precluding an origin by differentiation of basalt^17^. Chesner^17^ suggested that the source rocks of Toba magmas were metavolcanics and metasediments, and that a continental sedimentary source from Paleozoic metasedimentary basement^25^ is required. Similarly, Budd et al.^26^ suggested that Toba quartz crystals exhibit comparatively high δ^18^O values, up to 10.2‰, due to magma residence within, and assimilation of, local granitic basement.

In this study, two OTT samples were collected from an outcrop along the western margin (caldera wall) of Lake Toba (Fig. 1). Sample 1809–2 was collected ~ 100 m above sample 1809–1 on the same roadcut exposure during a field trip conducted by International Association of Volcanology and Chemistry of the Earth's Interior (IAVCEI) in 2018. The OTT samples are both strongly indurated to welded ignimbrite with fiamme. The samples are crystal rich, containing quartz, plagioclase, sanidine, biotite and amphibole with accessary minerals of Fe–Ti oxides, zircon, apatite and allanite. Sample 1809-2 was assumed to be YTT based on mineralogy (YTT quartz is pale whereas OTT quartz is typically pinkish; YTT has less biotite and has a lower proportion of crystals compared to OTT). However, the sample was determined to be OTT based on new U–Pb dating result in this study.

## Results

**Figure 3:**
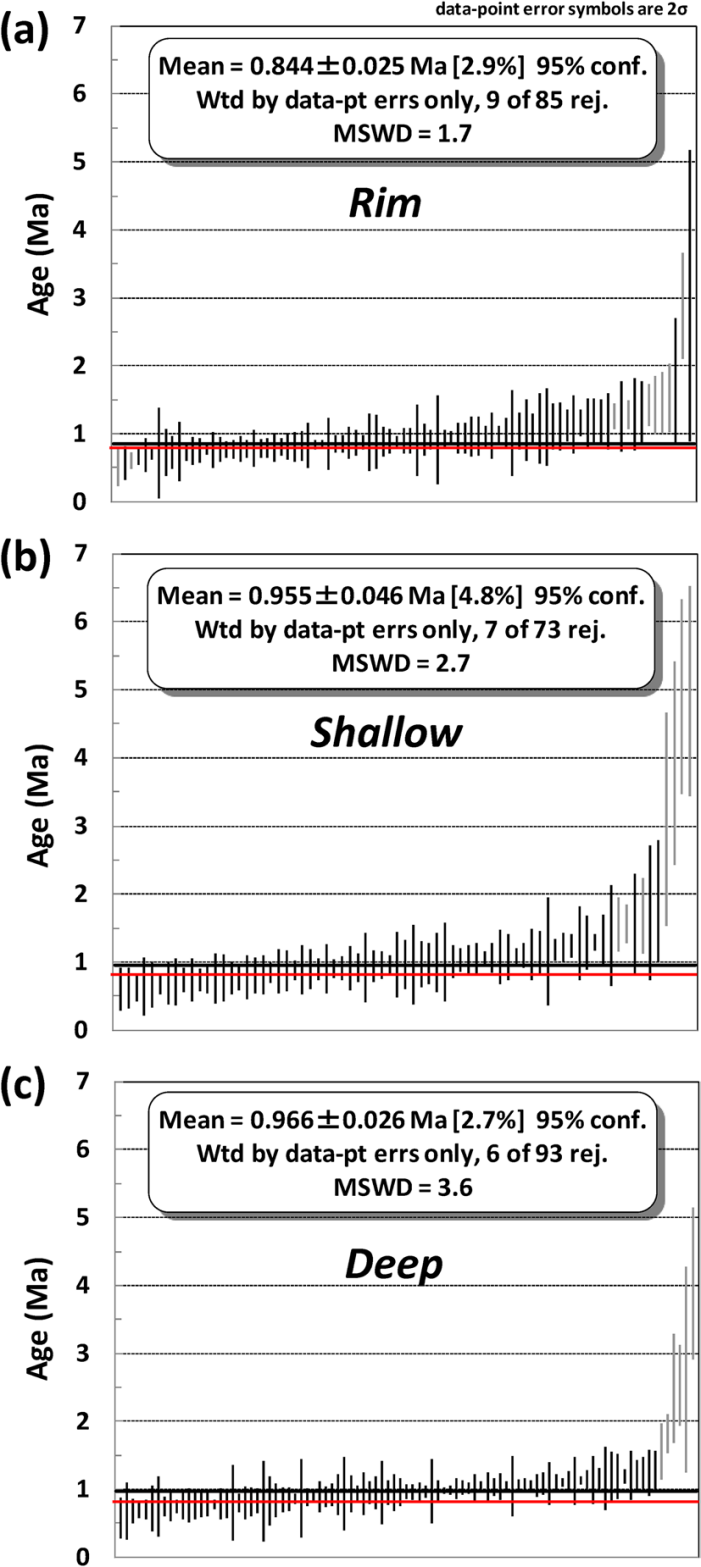
^238^U–^206^Pb age distributions for OTT zircons younger than 5 Ma. Individual grain ages with 2σ uncertainty are arranged from young to old. Bars in grey represent statistical outliers and were excluded. Black and red horizontal bars represent mean U–Pb ages and reported ^40^Ar/^39^Ar eruption ages (see text), respectively. (**a**) rim (< 8 μm in depth), (**b**) shallow (~ 9–18 μm in depth), (**c**) deep (~ 18–27 μm in depth). MSWD, mean square weighted deviation.

Ninety-nine zircon rims and 100 zircon interiors (shallow and deep sections) were analyzed for U–Pb isotopic ages by LA-ICP-MS. For some zircon crystals, both the rim and interior sections were analyzed to compare age difference within a single grain. The results are separately shown in Supplementary Tables S2–S4. Data with high (> 75%) common Pb contamination or high (> 70%) uncertainty were excluded for further interpretation because these data are less reliable. In total, the OTT zircon rims yielded a weighted mean age of 0.84 ± 0.03 Ma (95% confidence level; MSWD = 1.7; n = 76), shallow zircon interiors yielded an age of 0.96 ± 0.05 Ma (MSWD = 2.7; n = 66), and deep interiors yielded 0.97 ± 0.03 Ma (MSWD = 3.6; n = 87) (Fig. 3).

## Discussion

The eruption age for OTT is based on previously reported ^40^Ar/^39^Ar analyses; 789 ± 12 ka (2σ) on sanidine^27^, 799.7 ± 19.9 ka (2σ) on biotite^28^, and 798.8 ± 23.7 ka (2σ) on glass shards^4^. They are all in agreement with the cyclostratigraphic estimate of 788.0 ± 2.2 ka, using data from marine boreholes^3,28^. Therefore, I assume that the OTT erupted at ~ 0.8 Ma. An allanite Th–Pb crystallization age of 0.83 ± 0.04 Ma (2σ) reported for OTT^29^ is also in agreement with the interpreted eruption age.

All the youngest weighted mean ages from the three different OTT zircon depth sections overlap the eruption age of 0.8 Ma within 2σ uncertainty (Fig. 3), indicating that zircon crystallization continued just before the eruption. The 0.84 ± 0.03 Ma from the rim section is resolvably younger than the ages measured from zircon interiors, reflecting continued overgrowth of zircon crystallization. Moreover, the fact that the deep section age of 0.97 ± 0.03 Ma is identical to the shallow section age of 0.96 ± 0.05 Ma indicates that inheritance of older material is rare. In fact, there were three inherited zircons out of 151 analyzed zircons in OTT (i.e., zircon 1809-1-2-30 yielded a ~ 573 Ma rim, zircon 1809-1-40 was ~ 44 Ma and ~ 68 Ma in shallow and deep sections, respectively, and zircon 1809-2-1 yielded ~ 4 Ma and ~ 48 Ma in shallow and deep sections, respectively). These inheritance ages were omitted in calculated weighted mean ages.

The small proportion of inherited zircon in OTT is similar to results from the YTT. Reid and Vazquez^7^ suggested that the paucity of inherited zircon in YTT is notable because relatively old crustal material several kilometers-thick clearly played an important role in melt petrogenesis. They postulated crustal material was incorporated without significant entrainment of zircons and/or country rock zircons were nearly quantitatively resorbed during crustal melting, whose conditions are favorable in the lower crust. Similar assimilation conditions are also interpreted for OTT magma.

**Figure 4:**
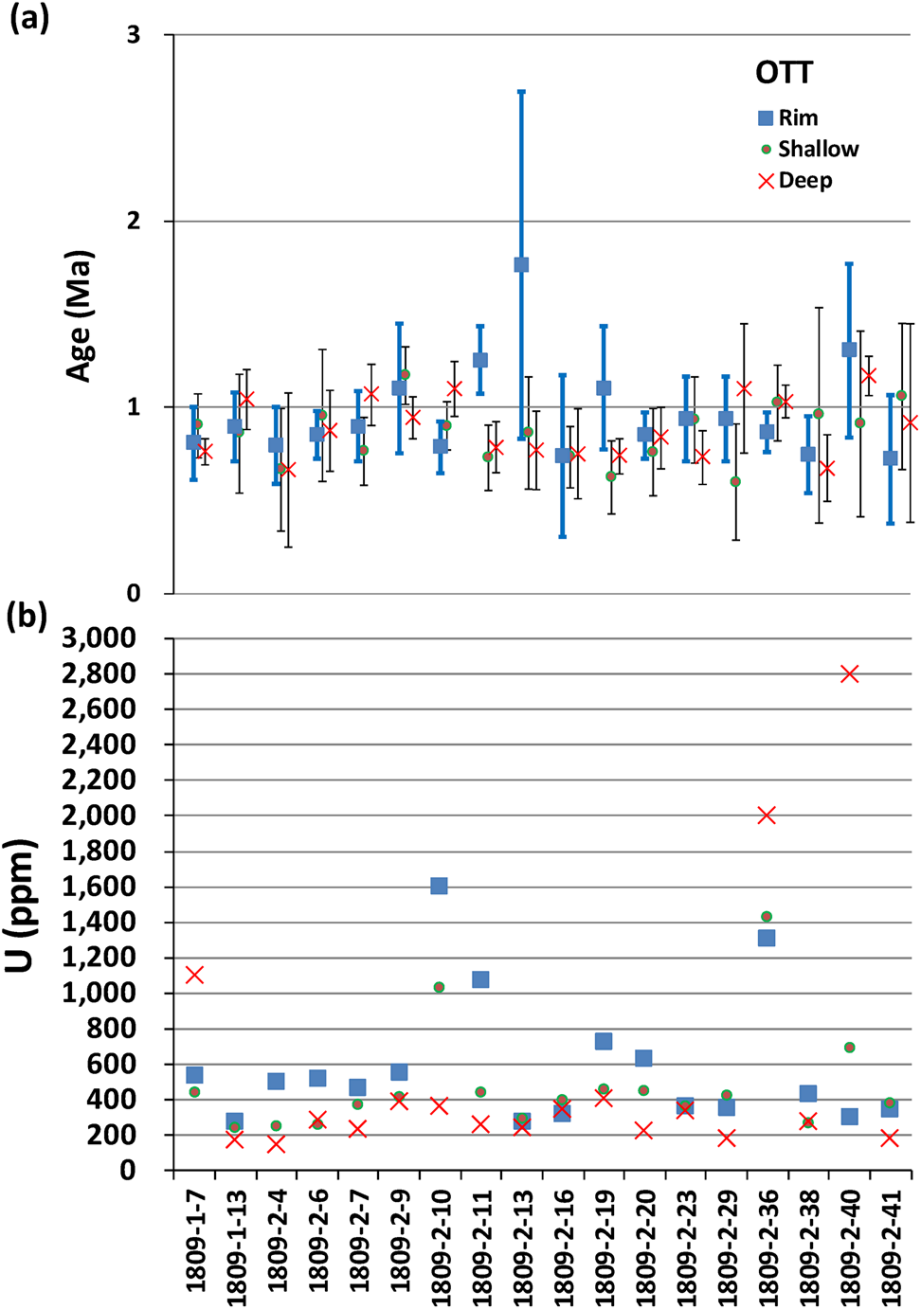
Comparison of (**a**) rim, shallow, and deep section ages with 2σ uncertainty and (**b**) U concentration (ppm) measured in a same OTT zircon grain. The uncertainty in U concentration (not shown) is assumed to be < 20%.

**Figure 5:**
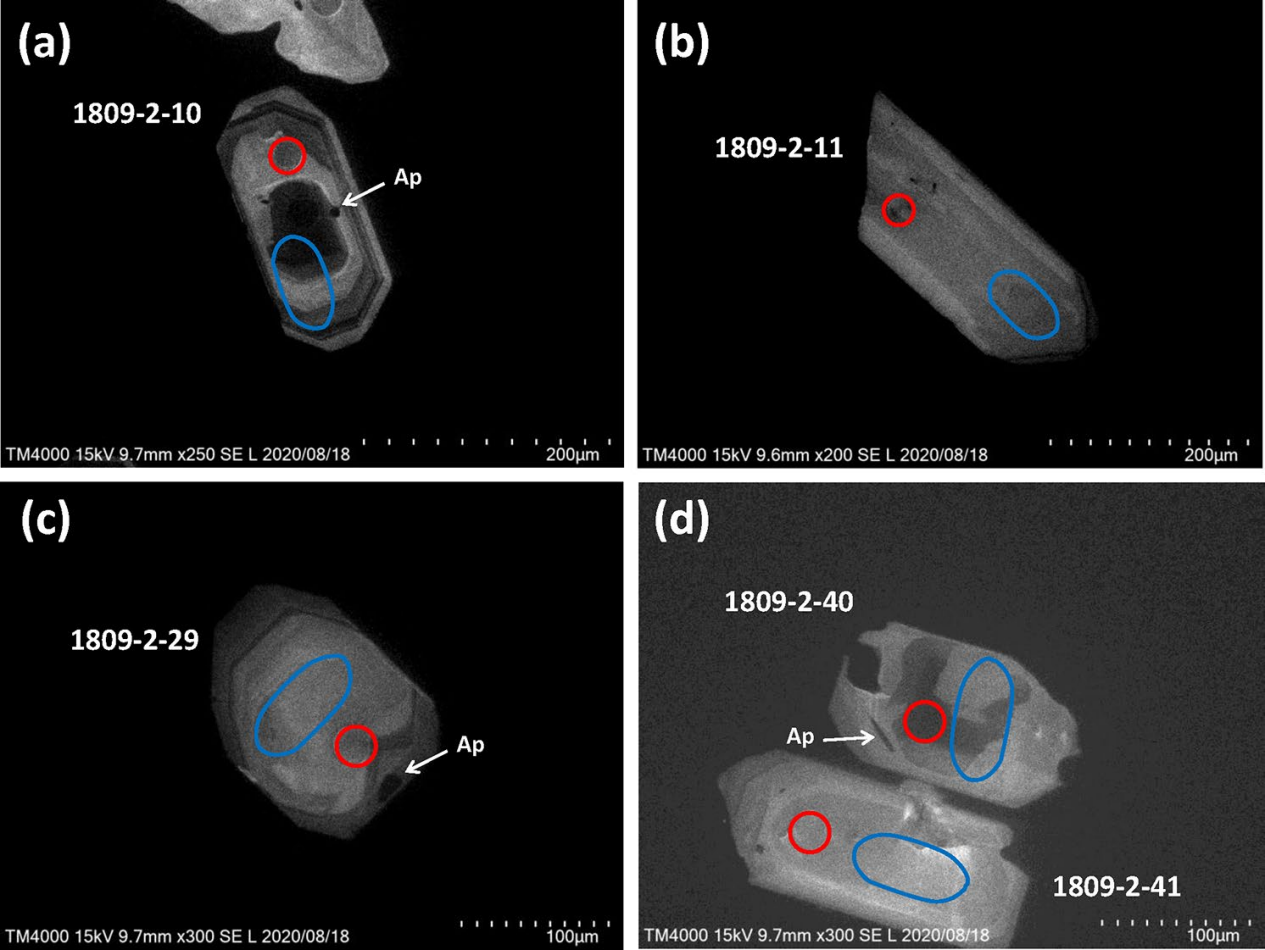
Representative cathodoluminescence (CL) images of OTT zircon polished to a depth of (**a**) ~ 19 μm, (**b**) ~ 15 μm, (**c**) ~ 16 μm, and (**d**) ~ 15 μm. Red circular and blue oval lines indicate spot (shallow and deep) and line-scan (rim) laser pit positions, respectively. CL images were obtained using a Hitachi TM4000Plus electron microscope. Polished depths were calculated by measuring the depths of laser spot before and after polishing using a KEYENCE VK-X 1000 laser scanning confocal microscope. Dark patches shown by arrows are apatite (Ap) inclusions.

Comparison of the ages from rim, shallow, and deep sections obtained on the same zircon grains is not straightforward to interpret (Fig. 4a). In general, zircon ages should become younger toward crystal surface but this trend is not statistically distinguishable due to the relatively low analytical precision of LA-ICP-MS analyses. For example, zircons 1809-2-10 and 1809-2-11 show younger and older rim ages than their interior ages, respectively. Cathodoluminescence (CL) image of 1809-2-10 (Fig. 5a) shows clear oscillatory zoning which is indicative of progressive and punctuated crystallization and consistent with its younger rim age. On the other hand, CL image of 1809-2-11 (Fig. 5b) show patchy sector zoning. Although it is difficult to explain the older rim age, the CL image may indicate a different growth history from zircon 1809-2-10. Zircon 1809-2-29 has a darker rim in CL than its interior (Fig. 5c), which corresponds with the higher uranium content in the rim (Fig. 4b). Similarly, zircon 1809-2-40 has a dark CL interior (Fig. 5d), which also yields higher uranium content (Fig. 4b).

The fact that some rim ages are as old as ~ 1.2 Ma (Fig. 3a) and some interior ages are as young as ~ 0.8 Ma (Fig. 3b,c) may indicate that zircons grew at different times in different parts of the magma reservoir due to intra-reservoir convection and remobilization processes, similar to YTT petrogenesis^26^.

Weighted mean ages of 1.19 ± 0.07 Ma and 1.20 ± 0.08 Ma are obtained taking the oldest 10 and 6 zircon ages from the deep section, respectively (Fig. 3c). From this, I interpret that OTT zircon crystallization started at ~ 1.20 Ma, coeval with the HDT eruption, and that the duration of OTT zircon crystallization prior to eruption suggests a magmatic residence time of > 400,000 years for OTT magma. This is comparable with the 500,000 years of YTT magmatism^7^. Although evidence for the vents or caldera margin associated with the OTT is scarce because the YTT presumably engulfed them^30^, the similar whole rock and zircon geochemistry of OTT and YTT^3,17,18,31,32^ (Fig. 2) and similarly long magmatic duration may indicate that they are similar in size. The commonly accepted caldera size of YTT is at least 2 times larger than OTT in dimensions (Fig. 1), whereas the actual caldera size of OTT might be larger than that shown in Fig. 1. New results from this study suggest the distribution of YTT might be overestimated, as I have obtained U–Pb ages that are consistent with OTT for an outcrop locality that was previously assumed to be YTT. The Pusuk Buhit Lava Flows, located near the sites of the OTT sampled in this study (Fig. 1), has a reported zircon U–Pb age of 0.91 ± 0.10 Ma (2σ)^33^, which also may indicate some of the OTT vents are located near Pusuk Buhit.

Finally, it is evident from this study and Matsu’ura et al.^13^ that LA-ICP-MS can be used to obtain information on the latest phase of zircon crystallization (material analyzed predominantly shallower than 5 μm depth), which is comparable to the spatial resolution of SIMS analyses^7,34^. LA-ICP-MS is more convenient compared to SIMS and when applied to sample surfaces, as in this study, it has the potential to yield robust U–Pb ages from the latest phase of zircon crystallization.

## Methods

Zircons from OTT samples (each ~ 1 kg) were separated using standard heavy liquid and magnetic separation techniques, yielding a few hundred zircons from each sample. Most zircons are euhedral to subhedral, including large (> 400 μm in length) and elongated zircons (Supplementary Fig. S1). Many zircons contain inclusions of glass and euhedral apatites of various size (Fig. 5). Most zircons exhibit oscillatory growth zoning in CL, although some are sector zoned (Fig. 5, Supplementary Fig. S1). Zircons were embedded in a PFA Teflon sheet and unpolished surface was targeted for analyses.

Zircon U–Pb dating was performed at the Central Research Institute of Electric Power Industry, using LA-ICP-MS (a Thermo Fisher Scientific ELEMENT XR magnetic sector-field ICP-MS coupled to a New Wave Research UP-213 Nd-YAG laser) with experimental conditions following Ito et al.^35^ and Ito and Danišík^36^ (Supplementary Table S1). Zircon interior ages were obtained as follows: A 30 μm laser beam with ~ 7 J/cm^2^ energy density and 10 Hz repetition rate was used to ablate the sample for 30 s following a 30 s background measurement. U–Pb (^238^U–^206^Pb) ages were obtained using the 10–20 s laser ablation data for shallow section, and using the 20–30 s data for deep section. Note that the first 10 s data were discarded due to signal instability. Because a 30 s laser ablation creates a pit of ~ 27 μm in depth in zircon (Supplementary Figs. S1, S2), I assumed that the shallow section U–Pb age is derived from material ablated between ~ 9 and 18 μm depths and ~ 18 to 27 μm depths for deep sections. Note that this procedure cannot exclusively sample age information at the targeted depths because mixing from shallower depths happens to some extent^37^.

In order to obtain zircon surface (or near surface) ages, the following conditions were also employed: a 40 μm laser beam with ~ 7 J/cm^2^ energy density, 5 Hz repetition rate and a horizontal scan speed of 2 μm/s for 20 s. In this condition, a shallow (maximum depth: ~ 8 μm) triangle-shaped pit (in profile view) approximately 80 μm in length was created (Supplementary Fig. S2). Therefore, > 80% in volume analyzed from the surface is shallower than 5 μm in depth. The surface (or rims) U–Pb ages were obtained using the 10–20 s laser ablation data.

Individual U–Pb ages were corrected for common-Pb using a modified ^207^Pb-based method^38^ using values from Stacey and Kramers^39^ and measured ^207^Pb/^206^Pb. The modified ^207^Pb method employs both Th/U and Pa/U partitioning (*f*_Th/U_ and *f*_Pa/U_, respectively) for correcting initial ^230^Th and ^231^Pa disequilibrium in the zircon-magma system. Measured *f*_Th/U_ with 20% uncertainty and an assumed *f*_Pa/U_ of 3.36 ± 0.40^38^ were used for all age calculations. Data with a high common Pb contamination (*f*_206_%) of > 75% were excluded for further analyses^35^ and the mean square weighted deviation (MSWD)^40^ is used as a statistical test of validity of weighted mean ages. Polished 91500 zircon^41^ was used as a primary standard with polished Plešovice^42^ zircon and unpolished Bishop Tuff^43^ zircon used as secondary reference materials.

U and Th concentrations were quantified by comparing counts of ^238^U and ^232^Th for the sample relative to the standard 91500, which is assumed to have homogeneous U and Th concentrations of 80 and 30 ppm respectively^41^, followed by a correction relative to NIST SRM 610 glass standard. The uncertainty of U and Th were not quantified but should be < 20% considering the measured U and Th concentrations of secondary standards (Plešovice and Bishop Tuff) (Supplementary Tables S2–S4) and NIST SRM 610 glass standard.

No down-hole isotopic (Pb/U, Th/U) fractionation correction was performed because data from the same depth range (or time span) was used for standards and unknowns in each analysis. Representative raw time-series U–Pb signal data are shown in Supplementary Figs. S3–S8.

The rim, shallow, and deep section U–Pb ages from the Plešovice zircon were between ~ 334 and 339 Ma, consistent with the reference value of 337 Ma (Supplementary Tables S2–S4). All the Bishop Tuff ages (~ 0.73 Ma) were slightly younger (~ 40,000 years younger) than the reference value of 0.77 Ma (Supplementary Tables S2–S4), which does not affect the main conclusions.

## Supplementary information

Supplementary file1

Supplementary file2

## Data Availability

Data are available in Supplementary information.
