# Supplementary material for: Magmatic history of the Oldest Toba Tuff inferred from zircon U–Pb geochronology
Source: Sci Rep. 2020 Oct 15;10:17506. doi: 10.1038/s41598-020-74512-z (PMC7566466; doi:10.1038/s41598-020-74512-z)
Supplement: Supplementary file 1 — Supplementary file1 [file 41598_2020_74512_MOESM1_ESM.pdf]

Magmatic history of the Oldest Toba Tuff inferred from zircon U-Pb geochronology

Hisatoshi Ito[1]

[1]*Nuclear Risk Research Center, Central Research Institute of Electric Power Industry, Chiba 270-1194, Japan*

Supplementary Information

Figure S1. Representative images of Oldest Toba Tuff (OTT) zircons.

Figure S2. Laser ablations on an OTT unpolished zircon (sample 1809-2-59) surface and their vertical profile.

Figure S3. Representative raw time-series U-Pb data for OTT rim analysis (sample: 1809-2-10L).

Figure S4. Representative raw time-series U-Pb data for Plešovice rim analysis (sample: P3-1-742L).

Figure S5. Representative raw time-series U-Pb data for Bishop Tuff rim analysis (sample: BST3-36L).

Figure S6. Representative raw time-series U-Pb data for OTT shallow and deep analyses (sample: 1809-2-10).

Figure S7. Representative raw time-series U-Pb data for Plešovice shallow and deep analyses (sample: P3-2-588).

Figure S8. Representative raw time-series U-Pb data for Bishop Tuff shallow and deep analyses (sample: BST2-31).

Supplementary Tables S1–S5 are available in a separate Excel file.

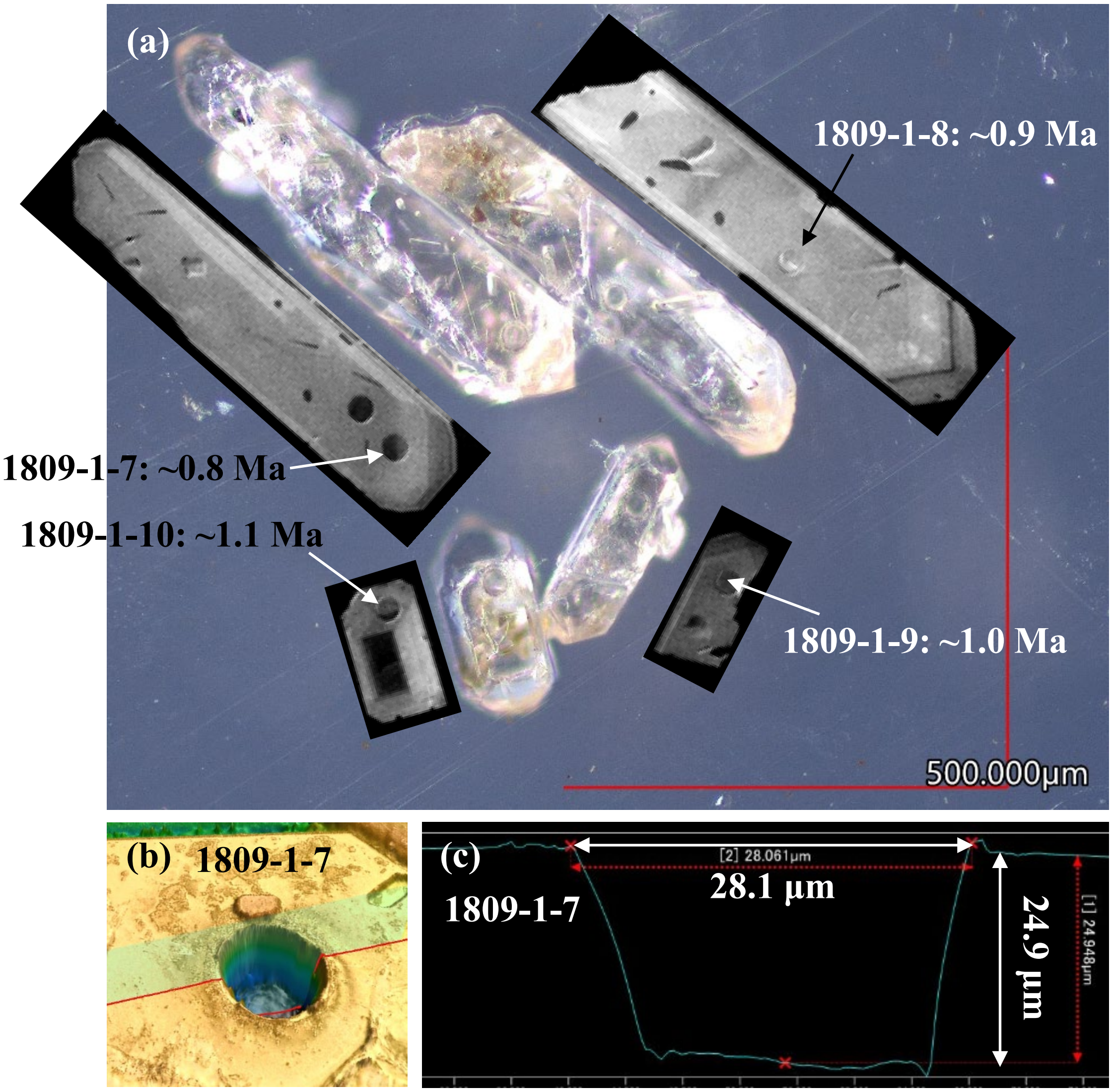

Figure S1. Representative images of Oldest Toba Tuff (OTT) zircons. (a) Zircons embedded in PFA Teflon. Cathodoluminescence (CL) polished zircon images are added to the original transmitted images of unpolished zircon. Sample names with U-Pb ages are shown. Laser ablation pits for U-Pb dating are indicated by arrows. (b) Close-up of the laser pit of sample 1809-1-7 (unpolished zircon). A red line is the profile line in (c). (c) Vertical profile of laser pit in sample 1809-1-7. Depth profile was obtained using a KEYENCE VK-X 1000 laser microscope.

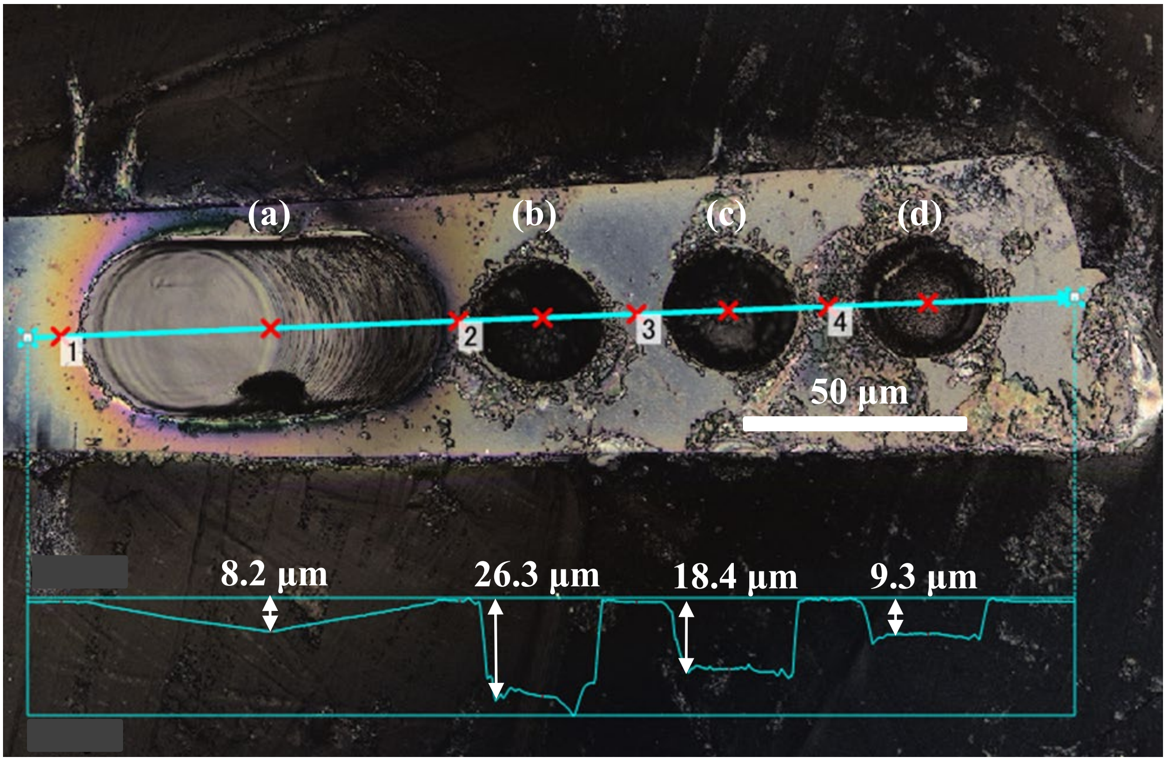

Figure S2. Laser ablations on an OTT unpolished zircon (sample 1809-2-59) surface and their vertical profile. (a) line scan with 40 µm laser beam (maximum depth ~8 µm), (b)–(d) 30 µm laser spot for 30 s (b), 20 s (c), and 10 s (d) ablations.

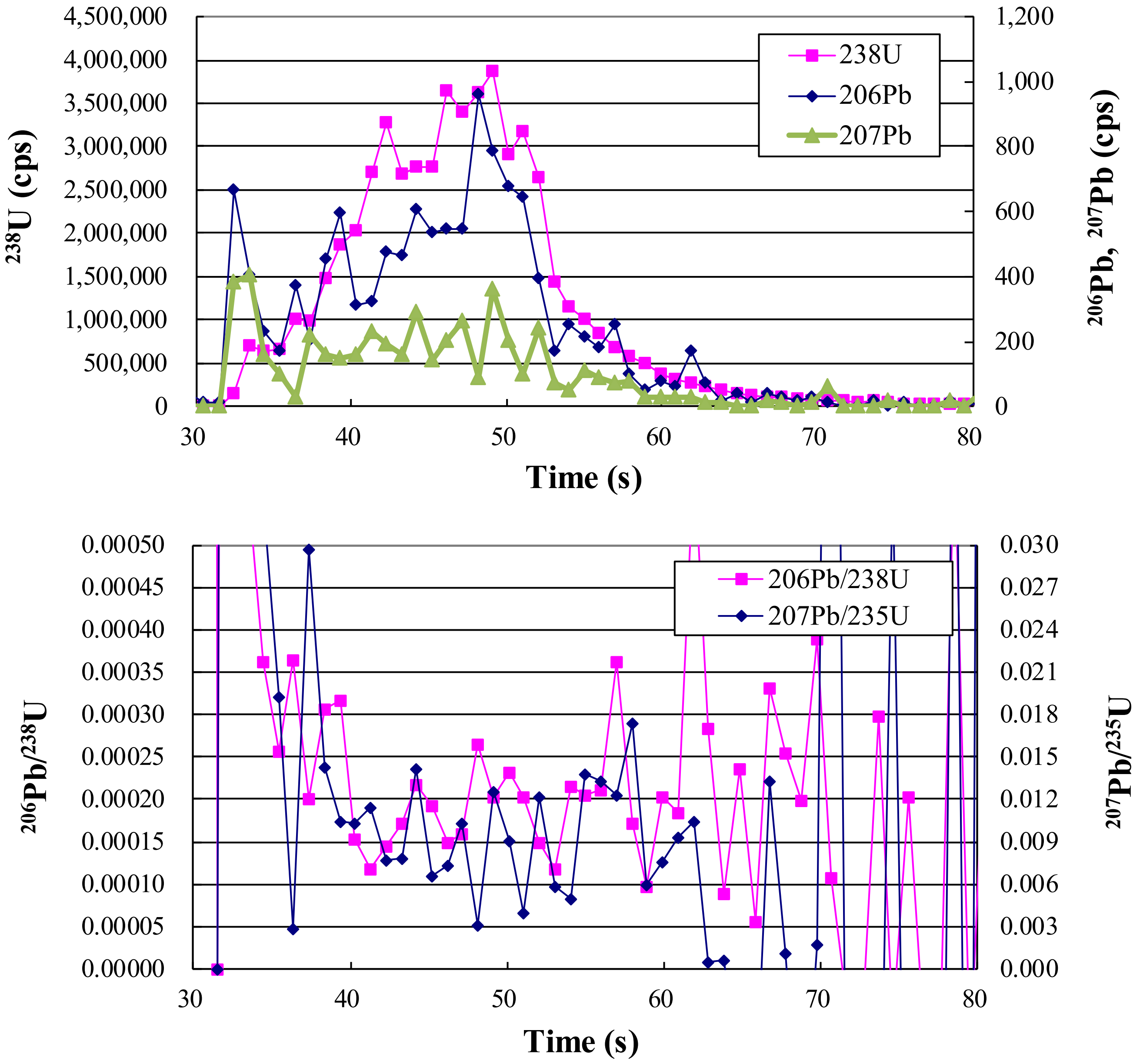

Figure S3. Representative raw time-series U-Pb data for OTT rim analysis (sample: 1809-2-10L). Signals from 40 to 50 s were used for age calculation.

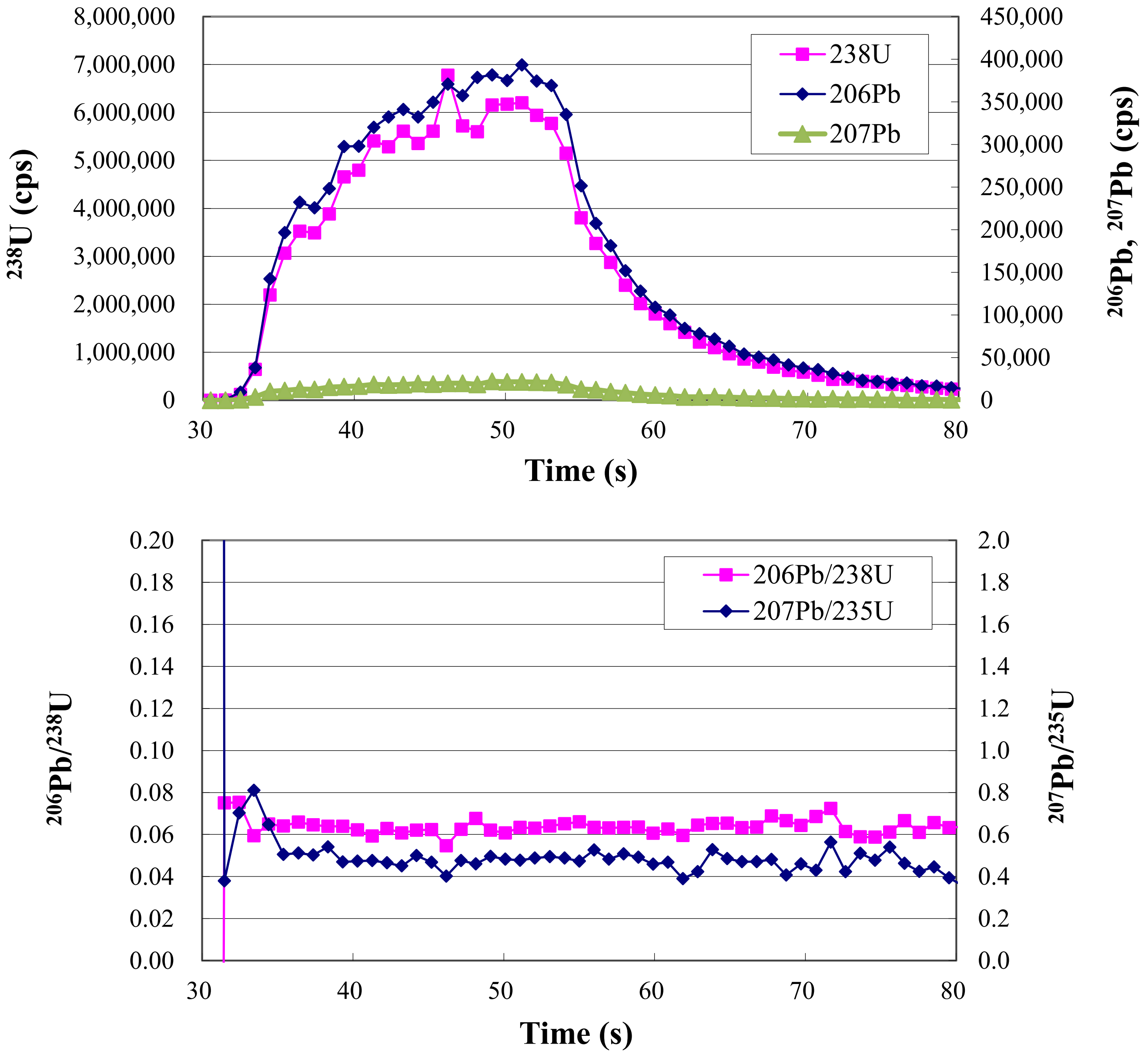

Figure S4. Representative raw time-series U-Pb data for Plešovice rim analysis (sample: P3-1-742L). Signals from 40 to 50 s were used for age calculation.

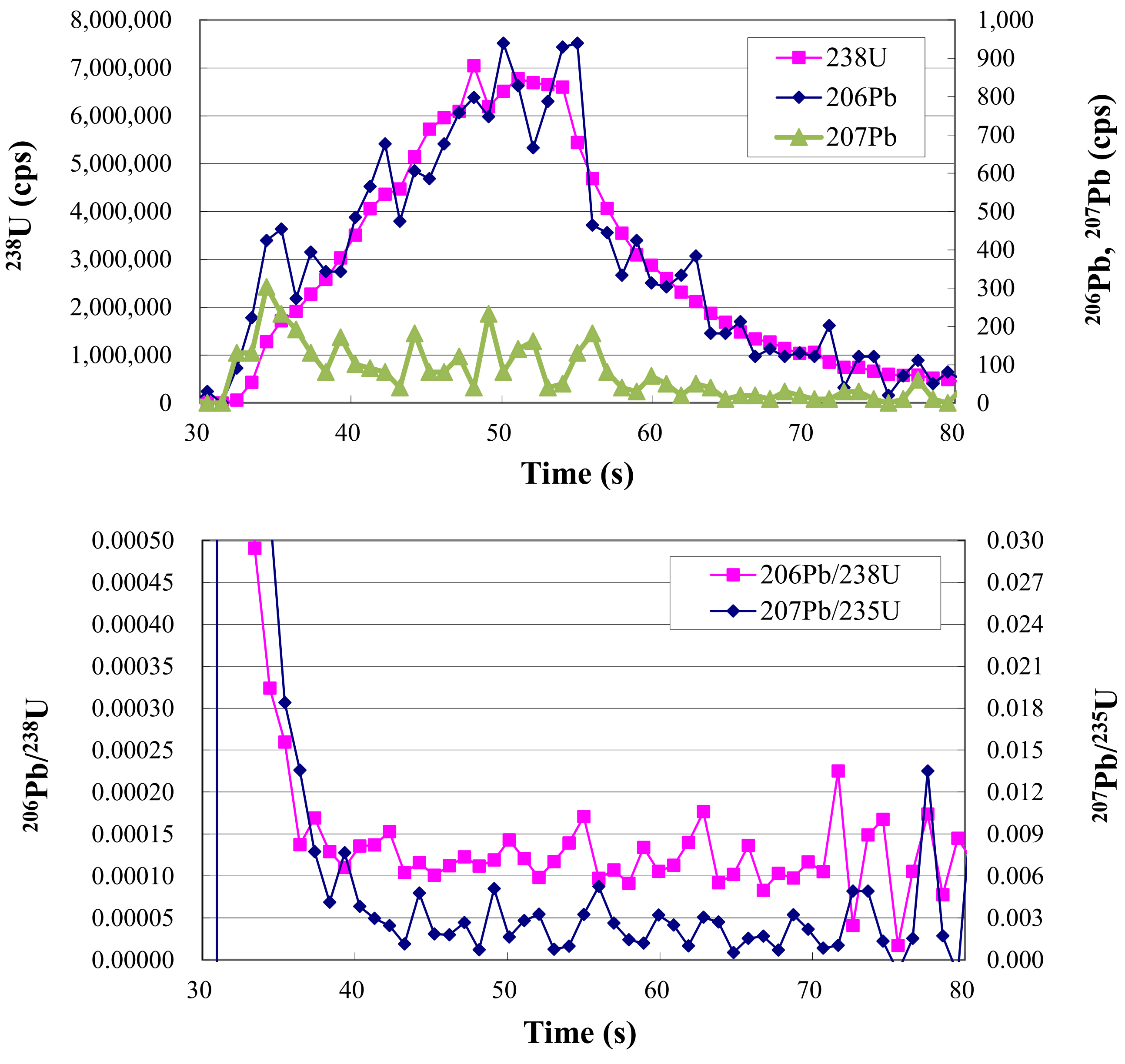

Figure S5. Representative raw time-series U-Pb data for Bishop Tuff rim analysis (sample: BST3-36L). Signals from 40 to 50 s were used for age calculation.

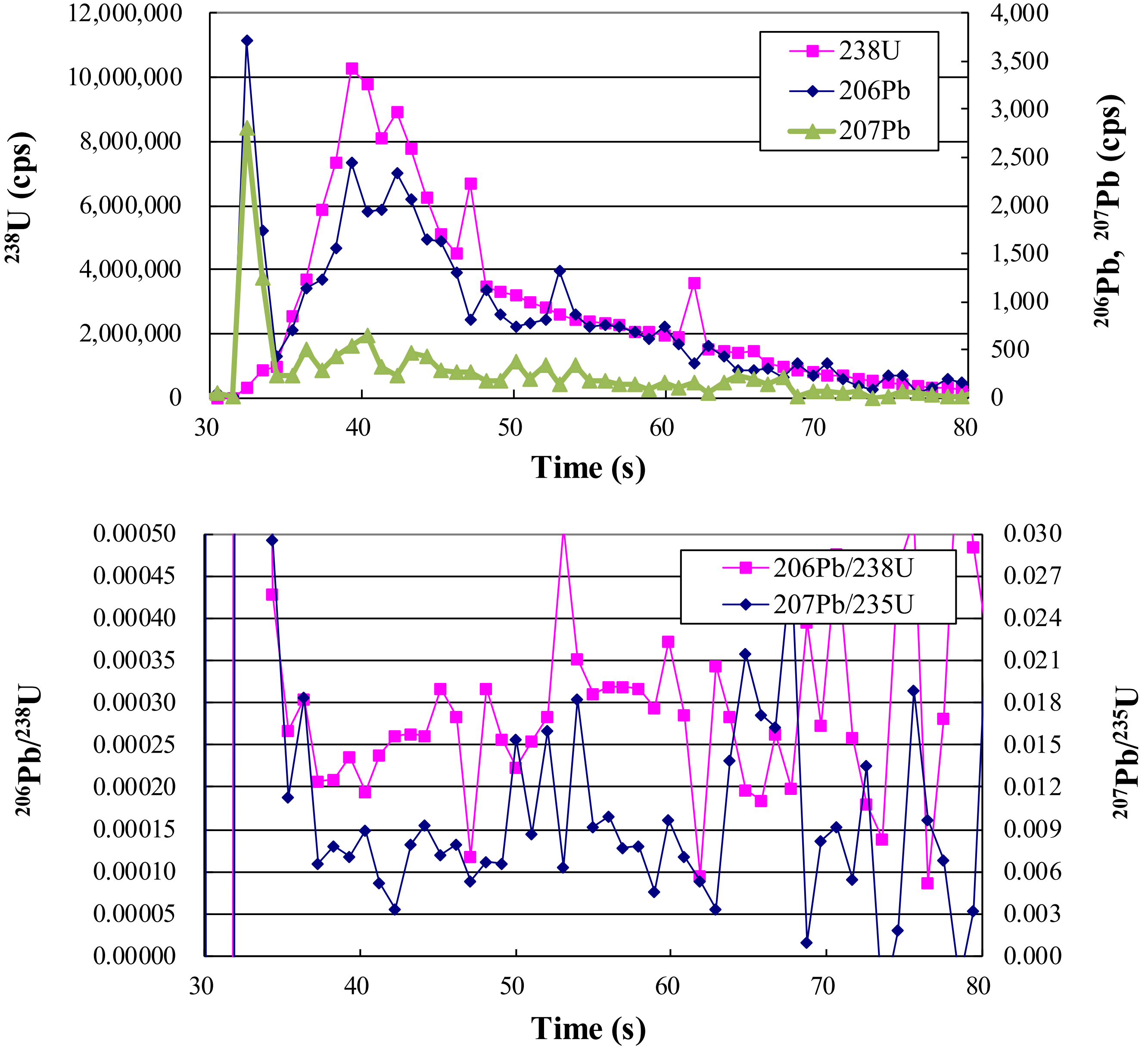

Figure S6. Representative raw time-series U-Pb data for OTT shallow and deep analyses (sample: 1809-2-10). Signals from 40 to 50 s and 50 to 60 s were used for shallow and deep age calculations, respectively.

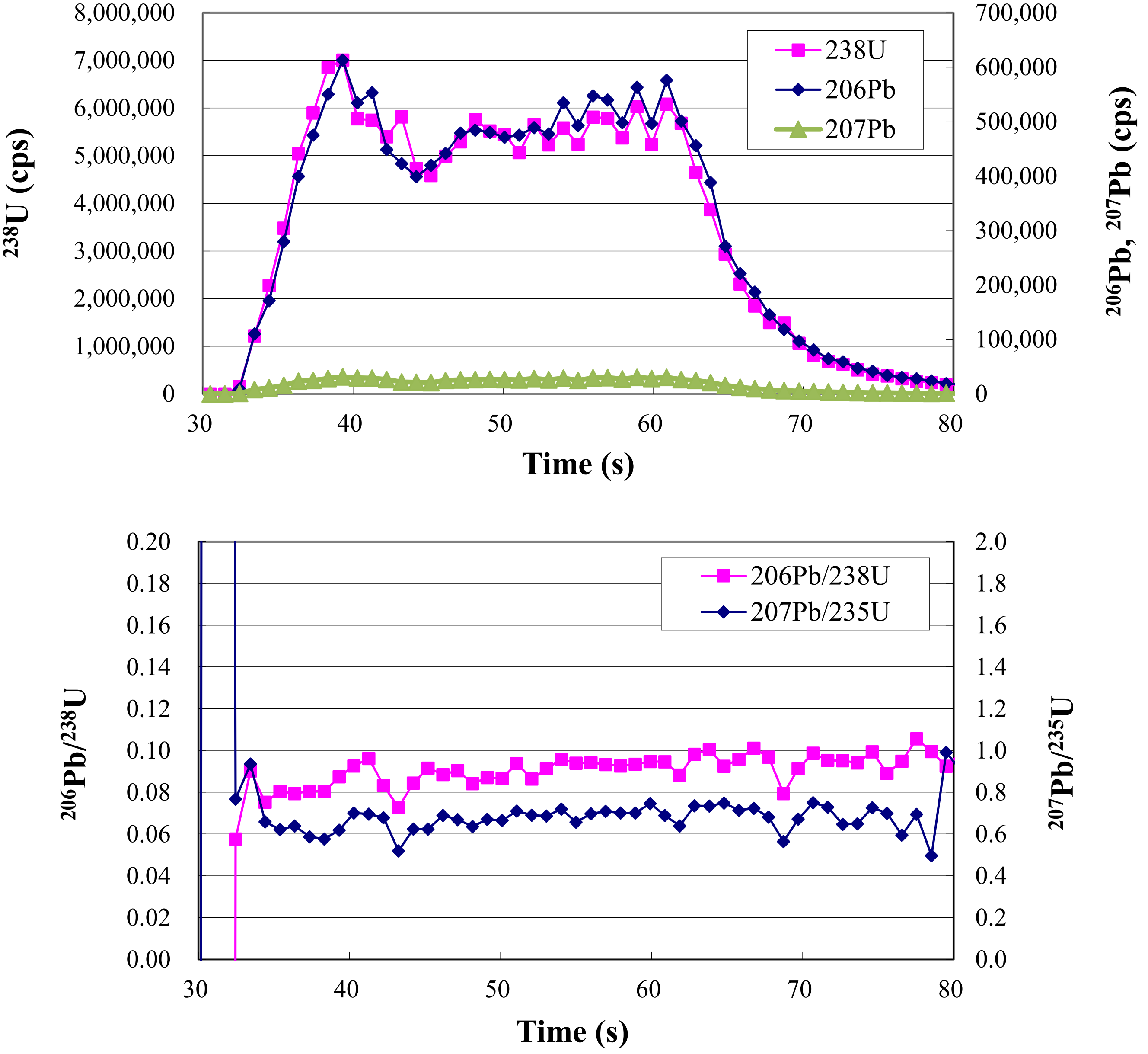

Figure S7. Representative raw time-series U-Pb data for Plešovice shallow and deep analyses (sample: P3-2-588). Signals from 40 to 50 s and 50 to 60 s were used for shallow and deep age calculations, respectively.

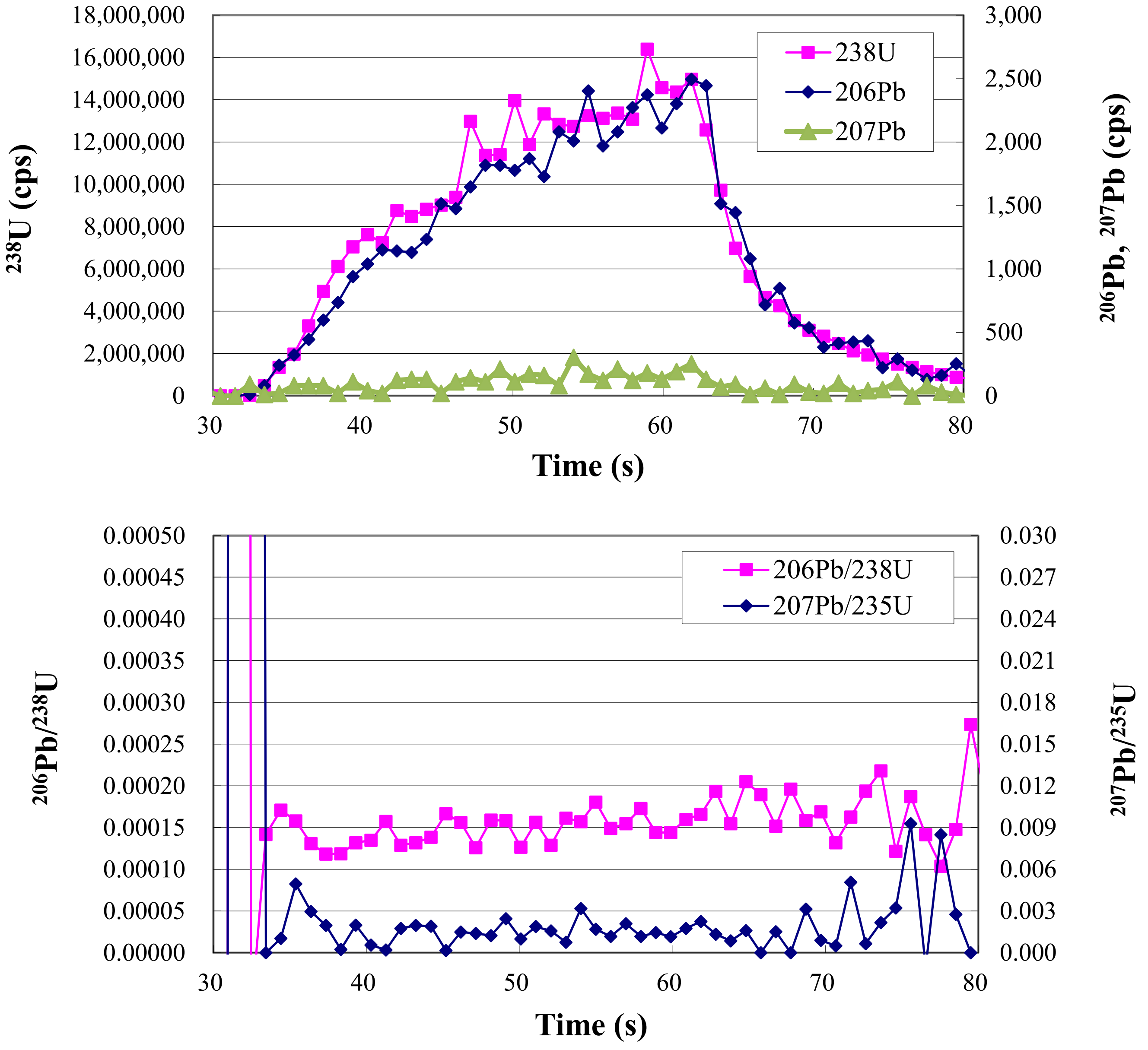

Figure S8. Representative raw time-series U-Pb data for Bishop Tuff shallow and deep analyses (sample: BST2-31). Signals from 40 to 50 s and 50 to 60 s were used for shallow and deep age calculations, respectively.
